# Editorial: Multi-omics approaches for decoding heterogeneity in cancer immunotherapy

**DOI:** 10.3389/fphar.2023.1324212

**Published:** 2023-11-03

**Authors:** Aimin Jiang, Ying Liu, Ouyang Chen, Zhigang Liu, Hongzhou Cai, Linhui Wang, Lin Qi

**Affiliations:** ^1^ Department of Urology, Changhai Hospital, Naval Medical University, Shanghai, China; ^2^ Department of Cell Biology, Duke University Medical Center, Durham, NC, United States; ^3^ Dongguan Key Laboratory of Precision Diagnosis and Treatment for Tumors, Dongguan, Guangdong, China; ^4^ Department of Urology, Jiangsu Cancer Hospital, Jiangsu Institute of Cancer Research, The Affiliated Cancer Hospital of Nanjing Medical University, Nanjing, Jiangsu, China; ^5^ Department of Orthopedics, The Second Xiangya Hospital, Central South University, Changsha, Hunan, China; ^6^ Hunan Key Laboratory of Tumor Models and Individualized Medicine, The Second Xiangya Hospital, Changsha, Hunan, China

**Keywords:** multi-omics, tumor microenvironment, tumor heterogeneity, cancer immunotherapy, personalized medicine

The emergence of cancer immunotherapy has brought about a significant revolution in the field of oncology (Hegde and Chen, 2020). This innovative approach offers new possibilities for treating a wide range of malignancies by leveraging the immune system’s power. Nevertheless, the effectiveness of such therapeutic strategies can be restricted by the inherent heterogeneity present within and between tumors. Consequently, deciphering this heterogeneity represents a critical challenge in optimizing the efficiency of immunotherapies. Tumor heterogeneity refers to the observed biological diversity among cancer cells residing in a single tumor or across different tumors. It arises from a variety of factors, including genetic variations, epigenetic modifications, transcriptional changes, and alterations in protein expression or metabolic profiles (Jia et al., 2022). This intricate interplay complicates efforts to elicit consistent and sustained responses to immunotherapy, as various cell populations may display different susceptibility to immune attack or exert diverse influences on the tumor microenvironment.

This is where the importance of multi-omics approaches comes into play. By incorporating genomics, transcriptomics, proteomics, metabolomics, and other “-omics” technologies, multi-omics provides a comprehensive view of the various biological layers present within tumors (Lee et al., 2022). It enables the characterization of genomic alterations, mRNA expression levels, protein abundance, and metabolic profiles across diverse cellular populations, allowing for a comprehensive understanding of the molecular landscape of carcinomas. Such high-resolution mapping facilitates the identification of distinctive molecular signatures linked to either response or resistance to immunotherapy. It also helps in elucidating the underlying mechanisms governing tumor-immune interactions and the discovery of novel targets for immunotherapy. Furthermore, temporal multi-omics analyses can track the dynamic changes occurring within tumors over time or in response to treatment, providing valuable insights into the evolution of tumor heterogeneity and its impact on treatment outcomes. In summary, multi-omics represents a powerful tool in unravelling the complexity of tumor heterogeneity, a critical factor in fully unlocking the potential of cancer immunotherapy. The knowledge gained from these investigations is expected to significantly contribute to personalized medicine and ultimately improve patient outcomes in the era of immunotherapy.

Given the vast amount of multi-omics data available and the advanced approaches for high pathway data analysis, this Research Topic has been thoroughly explored through 16 original research articles authored by a total of 137 individuals. These articles provide a comprehensive overview of the role played by multi-omics approaches in decoding tumor heterogeneity and enhancing the efficacy of immunotherapy.

Uveal melanoma (UVM) represents a primary intraocular malignancy that not only significantly impairs patients’ visual function and overall health but also poses a significant therapeutic challenge. The basement membrane (BM), critical in instigating and preserving diverse biological processes such as cell polarity, organ morphogenesis, and adult function, houses certain genes responsible for synthesizing basement membrane proteins which serve as valuable prognostic biomarkers across various cancer types. In their pioneering study, Li et al. adeptly employed multi-omics UVM datasets to develop an innovative risk assessment framework predicated on BM-associated genes. This system lays a robust theoretical groundwork for crafting precise, personalized treatment approaches. Immunotherapy appears particularly beneficial for high-risk group UVM patients, while those within the low-risk group enjoy enhanced survival benefits. Interestingly, *in vitro* assays established that inhibiting ITGA5 expression, a BM-related gene, effectively stymies the proliferation, migration, and invasive capabilities of UVM cells. In essence, the BM-related model proposed by Li et al. study demonstrates exceptional predictive prowess, markedly influencing patient prognosis and guiding individualized treatment strategies. Furthermore, this novel model paves the way for evaluating the effectiveness of pre-immune interventions.

Abundant empirical research has underscored the pivotal roles that cancer stem cells (CSCs) assume in driving and disseminating cancer. A study spearheaded by Chen et al. centered specifically on CSCs, identifying a selection of CSC marker genes predicated on scRNA-seq data procured from colorectal cancer samples. Through analyzing these markers’ expression profiles, the team singled out 29 CSC marker genes. Two distinctive phenotypes were identified and termed as CSC1 and CSC2. CSC2 owned a shorter disease specific survival or DSS and heightened oxidative stress response. Notably, drug sensitivity analyses suggested that CSC2 was more receptive to 44 chemotherapy drugs relative to CSC1. To bolster prognostication, the researchers devised a seven-gene prognostic model, adept at distinguishing high-risk from low-risk patients. Specifically, 14 chemotherapy drugs were found to be more beneficial for the high-risk group, while 13 were more effective for the low-risk group. Collectively, these insights enhance our comprehension of CSCs’ role in shaping the evolution and progression of colorectal cancer. Importantly, the seven-gene prognostic model shows promise as an indicator for predicting responsiveness to immunotherapy and chemotherapy, concurrently supplying valuable prognostic information for colorectal cancer patients. Given the global prevalence of colorectal cancer and the inherent heterogeneity-induced survival paradox in stage II/III CRC tumor biology, tumor progression is intimately linked with ferroptosis. As such, ferroptosis-related genes (FRG) may constitute novel biomarkers for predicting cancer prognosis. In their study, Wang et al. crafted a machine learning framework, which was consisted of 83 combinations of 10 algorithms to pinpoint the most robust and stable colorectal cancer model derived from FRG signatures. Strikingly, the FRG signature outperformed the clinicopathological features, and significantly correlated with BRAF mutation and microsatellite instability. Furthermore, the FRG signature was segregated into a high-risk group and a low-risk subgroup. Notably, high FRG signature corresponded to poorer prognosis across all datasets. Crucially, sensitivity analysis affirmed the FRG signature as a significant prognostic indicator, underscoring its potential in guiding clinical decision-making and facilitating personalized therapy for stage II/III colorectal cancer patients.

Recent studies have unveiled the pivotal role that microbes assume in the onset, evolution, metastasis, and treatment response of diverse tumor types. This influence is especially pronounced in the context of the tumor microenvironment and immune response. A multitude of research has demonstrated that the intratumor microbiome can shape the progression, metastases, prognosis, and immunotherapy outcomes in cancer patients. This modulation occurs via control of oxidative stress, Toll-like receptor-mediated immune response, and tumor cell metabolism, which engages several signaling pathways including mTOR, STAT3, Wnt, and MAPK. However, current understanding falls short of comprehensively elucidating the relationship between the intratumor microbiome and clinicopathological features, prognosis, heterogeneity of the tumor microenvironment, and therapeutic response in pancreatic cancer (PC). In a study spearheaded by Zhang et al., 26 prognostic genera associated with PC were identified. The PC samples were categorized into two microbiome-related subtypes: Mcluster A and B. Patients in Mcluster B exhibited poorer prognosis, higher TNM stage, and pathological grade compared to those in Mcluster A. Immune analysis exposed significantly elevated levels of infiltrated CD8^+^ T cells, M1 and M2 macrophages, cancer-associated fibroblasts, myeloid dendritic cells, neutrophils, regulatory T cells, and activated mast cells in Mcluster B. Conversely, patients in Mcluster A were more likely to benefit from CTLA-4 blockers and exhibited heightened sensitivity to several agents including oxaliplatin, and epirubicin. Moreover, a microbe-derived model was devised to predict the outcome in PC, with ROC curves illustrating this model’s high predictive performance. Single-cell analysis identified the presence of monocytes/macrophages, endothelial cells, and fibroblasts along with cancer cells within the PC tumor microenvironment. Notably, LIPH and LAMA3 displayed relatively higher expression in cancer cells and neutrophils. Collectively, these insights offer new perspectives on outcome assessment and therapeutic strategies for PC.

The 5-year survival rate for patients with advanced-stage gastric cancer (GC) remains disappointingly low. Studies have proposed a role for tryptophan metabolism in cancer progression, implicating it in eliciting immunosuppressive responses and bolstering the malignancy of cancer cells. Thus, unraveling the roles of tryptophan and its metabolism is key to comprehending the molecular mechanisms implicated in GC evolution. In a recent study spearheaded by Luo et al., public datasets were employed to identify genes associated with tryptophan metabolism. Single sample gene set enrichment analysis, and correlation analysis were utilized to this end. Consequently, two molecular subtypes linked with tryptophan metabolism were pinpointed. The first subtype, coined C1, exhibited a more favorable prognosis than the second subtype, C2. This observation was accompanied by an uptick in CD4 positive memory T cells and activated dendritic cells (DCs) within the tumor microenvironment, along with a suppression of M2-phenotype macrophages. Furthermore, immune checkpoint activity was detected to be downregulated in the C1 subgroup. To construct a prognostic risk model, eight pivotal genes were identified as critical to predict the prognosis of GC patients. In sum, this study underscores the potential utility of tryptophan metabolism-associated genes in forecasting the prognosis of GC patients. The risk model devised in this study demonstrated remarkable precision in predicting survival outcomes in GC patients.

Hepatocellular carcinoma (HCC) represents an assertive form of liver cancer typically diagnosed at advanced stages, thereby contributing to high mortality rates. Hence, the discovery of new biomarkers for early detection and patient outcome improvement is imperative. Efferocytosis, a sophisticated process wherein one cell engulfs another, involves diverse immune cells like macrophages, dendritic cells, and natural killer (NK) cells. This process, intriguingly, plays a dual role in tumor development—sometime fostering, other times inhibiting tumorigenesis. Nevertheless, our understanding of efferocytosis-related genes’ (ERGs) role in HCC progression remains incomplete, particularly their influence on immunotherapy and targeted drug treatments. In a recent study helmed by Xu et al., a risk model predicated on six ERGs was designed, leading to the identification of two distinct HCC subtypes corresponding to these genes. The identified subtypes exhibited significant disparities in the tumor immune landscape and prognosis, accentuating efferocytosis status’s importance in HCC. Collectively, this investigation illuminates the critical role of efferocytosis in HCC and lays a solid groundwork for further exploration into this disease’s progression and treatment response. It holds potential in shaping clinical decision-making in HCC management. Additionally, 5-FU, a traditional chemotherapeutic drug utilized in various cancers, has been demonstrated to elicit an effective response not only by targeting cancer cells but also by stimulating an anti-tumor immune response mediated by the STING pathway within the cancer cells themselves. The cGAS-STING pathway activation by 5-FU treatment instigates local production of type I interferons (IFNs), implying that 5-FU could trigger IFN production specifically in the tumor microenvironment, even in cancers traditionally unaddressed with 5-FU. In a study conducted by Gu et al., HCC subtypes were classified based on their 5-FU sensitivity. The findings corroborated the observed prognostic disparities in HCC and underscored the heterogeneity of genomic variations, the tumor immune microenvironment, and pathological pathways. Additionally, the study devised an independent prognostic risk regression model incorporating five 5-FU-related genes, promoting individualized HCC monitoring advancement. In conclusion, these studies affirm 5-FU’s crucial role in bolstering anti-tumor immunity and offer valuable insights into this drug’s potential application in HCC treatment.

We have gathered three research papers on breast cancer for our Research Topic analysis. One crucial mechanism that has been widely recognized as a natural defense against tumors is cellular senescence. Oncogene-induced senescence (OIS), which involves the activation of proto-oncogenes or the deactivation of tumor suppressor genes, leads to cell growth arrest. Notably, Huang et al. conducted a comprehensive study using both in-silicon and experimental approaches and categorized triple-negative breast cancer (TNBC) into two subtypes, namely, TNBCSASP1 and TNBCSASP2. They based this classification on the set of genes associated with the senescence-associated secretory phenotype. Unfortunately, the TNBCSASP1 subtype displayed a poor prognosis. It was found to exhibit immunosuppression, with reduced activity in immune-related signaling pathways and limited infiltration of immune cells. The likelihood of TP53 and TGF-β pathways mutations contributing to the unfavourable prognosis of the TNBCSASP1 subtype was suggested. Additionally, the authors uncovered FAM3B as a key biomarker influencing the prognosis of patients with TNBC. In TNBC, FAM3B expression was lower than in normal breast tissue. Survival analysis demonstrated that patients with high FAM3B expression had significantly shorter overall survival rates. Unfortunately, due to the lack of effective treatments, triple-negative breast cancer has an extremely poor prognosis. Metabolic reprogramming is a fundamental aspect of tumorigenesis, cancer diagnosis, prognosis, and treatment. Li et al. conducted a fascinating study in which they identified two metabolically distinct subtypes of TNBC. The C1 subtype exhibited high expression of immune checkpoint genes and immune and stromal scores, suggesting sensitivity to PD-1 inhibitors. Conversely, the C2 subtype exhibited significant variation in carbohydrate, lipid, and amino acid metabolism pathways. Importantly, C2 lacked immune signatures, displayed late pathological stage, low immune infiltration, and correspondingly had a poor prognosis. C2 also had a high mutation frequency in PIK3CA, KMT2D, and KMT2C, and exhibited significant activation of the PI3K and angiogenesis pathways. In conclusion, the authors successfully identified two TNBC subtypes with distinct metabolic characteristics, offering valuable insights into TNBC heterogeneity and providing a theoretical foundation for potential therapeutic strategies. Among the various types of breast cancer, estrogen receptor (ER) breast cancer is the most prevalent, characterized by the expression of estrogen receptors. Globally, it affects approximately 2.26 million women. Khan et al. conducted a study that identified differentially expressed genes and isoform switching between ER-positive and triple-negative breast cancer samples. Specifically, they pinpointed six genes predominantly associated with ER-positive breast cancer, as well as a novel set of ten genes not previously reported in ER-positive breast cancer. Additionally, alternative splicing and subsequent isoform usage in genes related to the immune system were identified. This study sheds light on the differential isoform usage occurring in cancer cells and its potential implication in immunosuppression through dysregulation of CXCR chemokine receptor binding, iron ion binding, and cytokine activity.

Copper, an indispensable mineral vital for enzyme activity and transcription factor function, can trigger proteotoxic stress and a unique form of cell death known as cuproptosis when in excess, due to the accumulation of lipoylated dihydrolipoamide S-acetyltransferase (DLAT), linked with the TCA cycle. Cuproptosis, implicated in cancer progression, presents a promising therapeutic target. In their study, Kuang et al. utilized multi-omics datasets and extensive *in vitro* experiments to probe the biological and clinical significance of cuproptosis in lung adenocarcinoma (LUAD). Employing lasso regression analysis, they developed a cuproptosis-related signature (CRS) based on 24 specific genes. According to findings, high-risk CRS patients portended poorer prognosis in both TCGA-LUAD and GSE31210 datasets. Further enrichment analysis disclosed that copper proliferation primarily transpired via chromosome-related pathways, cell cycle regulation, DNA replication, and G2M checkpoint activation. Additionally, differences in macrophage levels were observed between low and high CRS groups through immuno-infiltration analysis. Crucially, these cuproptosis-related genes might serve as potential prognostic predictors and immunotherapy effectiveness indicators for LUAD patients by influencing chromosome-related pathways and macrophage activity. Concurrently, Cong et al. study employed machine learning algorithms such as GBM, lasso, xgboost, SVM, random Forest, and Decision Trees to construct a novel risk stratification system based on fatty acid metabolism (FAM)-related signatures. Lower FAM-related pathway scores were noted in LUAD samples. Three molecular subtypes C1, C2, and C3 were defined, with differential prognostic analysis revealing the most favorable prognosis in C1 subtype, followed by C2, and the worst prognosis in C3 subtype. The C3 subtype also displayed lower levels of immune infiltration. A risk score model was developed using 12 key identified genes where high-risk score patients demonstrated significantly lower survival rates. Conversely, the low-risk score group showed higher immune scores and increased expression of immune checkpoint genes. Moreover, high-risk score patients were more likely to benefit from six anti-cancer drugs screened in this study. In essence, these studies offer valuable insights into the role of cuproptosis and fatty acid metabolism in LUAD’s prognosis and treatment. They underscore the potential utility of cuproptosis-related genes and fatty acid metabolism-related signatures in predicting patient outcomes and informing therapeutic strategies, including immunotherapy and targeted drug interventions.

The amplified cardiovascular event risk experienced by cancer patients presents a substantial concern. Extensive research suggests that cancer survivors are more prone to cardiac complications, including myocardial infarction, heart failure, and arrhythmias, relative to the general populace. Yuan et al. recent study illuminates that distinct monocyte-derived biomarkers harbor significant promise in prognosticating cancer outcomes and acute myocardial infarction (AMI). The researchers applied an innovative formula to examine mRNA levels in clinical samples from AMI and cancer-diagnosed patients, leading to the construction of a novel risk score based on expression profiles. By classifying patients into high-risk and low-risk groups according to the median risk score, noticeably poorer overall survival rates were observed among high-risk patients within the cancer cohorts, as corroborated by Kaplan-Meier analysis. Key to note is the concurrent activation of the Notch signaling pathway, potentially shedding light on shared high-risk factors affiliated with both AMI and cancer. Moreover, the researchers confirmed the differential expression of these genes in cell lines and clinical samples, further reinforcing their relevance as potent biomarkers. Crucially, these findings highlight the potential utility of shared biomarkers in accurately predicting patient outcomes for both cancer and AMI.

Serine protease inhibitor clade E member 1 (SERPINE1), or PAI-1, is a pivotal modulator of the plasminogen activation system, with roles extending beyond mere plasminogen regulation. Implicated in numerous physiological processes, SERPINE1’s multifaceted functions hint at its potential contribution to diverse disease processes. In an effort to demystify the role of SERPINE1, Li et al. conducted an exhaustive analysis across multiple cancer types. The study unveiled dysregulated SERPINE1 expression in cancer cells, noting enrichment in endothelial cells and fibroblasts. This irregularity can be partially attributed to copy number amplification and reduced DNA promoter methylation. Crucially, heightened SERPINE1 expression was linked with adverse prognosis in 21 disparate cancer types. Further scrutiny using gene set enrichment analysis (GSEA) presented SERPINE1 as a player in immune response regulation and tumor malignancy. Correlations were drawn between SERPINE1 expression, immunoregulator expression, and immune cell infiltration, suggesting a potential role for SERPINE1 in immunosuppression. Intriguingly, associations were also found linking SERPINE1 expression with tumor mutation burden (TMB), microsatellite instability (MSI), responses to immunotherapy, and drug sensitivity across various cancers. Collectively, this study emphasizes the anomalous expression of SERPINE1 in numerous cancer types and its consequent implications for cancer immunity and tumor behavior. Such findings furnish invaluable insights that could steer the development of personalized cancer treatments meticulously tailored to individual patients.

Metastatic castration-resistant prostate cancer (mCRPC), an intensely aggressive prostate cancer stage, exhibits progression largely driven by non-mutational epigenetic reprogramming. Super enhancers (SE), a category of epigenetic elements, are implicated in myriad tumor-promoting signaling pathways, though the precise modus operandi of SE mediation in mCRPC remains enigmatic. Zeng et al. employed a CUT&Tag assay on mCRPC C4-2B cell lines to identify SE-associated genes and transcription factors. Merging these overlapping genes, or SE-related DEGs, they developed a recurrence risk prediction model. A time-dependent receiver operating characteristic (ROC) curve analysis affirmed the robust predictive capacity of their risk score system at the 1-year (0.80), 3-year (0.85), and 5-year (0.88) stages. In sum, their study offers a comprehensive comprehension of the SE element landscape and related genes in mCRPC and furthers discourse on potential clinical implications and transitions to clinical practice.

Bladder cancer, a prevalent urologic malignancy associated with significant morbidity and mortality, presents varying patient response rates to the promising potential of immunotherapy. The process of glycosylation, characterized by sugar molecule attachment to proteins or lipids, has been implicated in tumor development and immune regulation. Yet, the clinical implications and understanding of its role in bladder cancer remain underdeveloped. Liu et al. sought to bridge this knowledge gap by conducting a multi-cohort study of bladder cancer patients, developing a unique risk scoring system cantered on glycosylation-related genes. In particular, the authors segmented the training cohort into two clusters based on distinct patterns of glycosylation-related gene expression. Prognostic analysis unveiled worse survival outcomes for Cluster 2 than Cluster 1. Cluster 2 also showcased elevated levels of tumor microenvironment-immune cells and amplified activity in crucial phases of the cancer immune response cycle. To corroborate their findings, a standalone prognostic risk score was developed, aiding in the construction of a reliable prognostic prediction nomogram. Patients with high glycosylation risk scores demonstrated an increase in tumor immune cell infiltration, higher enrichment scores within immune related pathways. Conversely, patients with lower risk scores exhibited minimal immune cell infiltration and tended towards a luminal subtype. These findings held true even in real-world examination, as evidenced by the Xiangya cohort. In essence, the study underscores that a multi-omics glycosylation score derived from these identified genes can reliably elucidate bladder cancer heterogeneity and predict immunotherapy effectiveness and molecular subtypes. Such pivotal insight empowers refined individual treatment decisions for bladder cancer patients.

Tumorigenesis, a multifaceted process marked by the perturbation of assorted genetic and non-genetic mechanisms that amass over a duration, is characterized by the inherent genomic instability of neoplastic cells. Such unstable features often yield serendipitous tumorigenic events throughout disease progression. These stochastic occurrences play a crucial role in molding an eclectic immune microenvironment—spatially or temporally—which introduces a degree of heterogeneity. The introduction of multi-omics profiling technologies has heralded a transformation in our comprehension of the labyrinthine and heterogeneous character of the Tumor Microenvironment (TME) at unparalleled resolution. These advanced multi-omics modalities offer unmatched granularity in deciphering the orchestrated modifications in composition and status of immune and stromal components within the TME. This results from cancer treatment responses, thus enabling a comprehensive and nuanced insight into cancer progression and immunotherapeutic outcomes.
